# Assessing the health literacy of caregivers in the pediatric intensive care unit: a mixed-methods study

**DOI:** 10.3389/fped.2023.1308673

**Published:** 2023-12-21

**Authors:** Anireddy R. Reddy, Anushree K. Doshi, Allison Mak, Judy A. Shea, Joana T. Fardad, Jiwon Moon, Paula Hu, Annery G. Garcia-Marcinkiewicz

**Affiliations:** ^1^ Department of Anesthesiology and Critical Care Medicine, Children’s Hospital of Philadelphia, Philadelphia, PA, United States; ^2^ Department of Anesthesiology and Critical Care, The University of Pennsylvania Perelman School of Medicine, Philadelphia, PA, United States; ^3^ Leonard Davis Institute of Health Economics, University of Pennsylvania, Philadelphia, PA, United States; ^4^ Department of General Surgery, Children’s Hospital of Philadelphia, Philadelphia, PA, United States; ^5^ Department of Medicine, Perelman School of Medicine and University of Pennsylvania, Philadelphia, PA, United States

**Keywords:** health literacy, pediatrics, intensive care, mixed-methods, implementation science

## Abstract

**Background:**

Limited health literacy is associated with increased hospitalizations, emergency visits, health care costs, and mortality. The health literacy levels of caregivers of critically ill children are unknown. This mixed-methods study aims to quantitatively assess the health literacy of caregivers of children admitted to the pediatric intensive care unit (PICU) and qualitatively describe facilitators and barriers to implementing health literacy screening from the provider perspective.

**Methods:**

Caregivers of patients admitted to our large, academic PICU (between August 12, 2022 and March 31, 2023) were approached to complete a survey with the Newest Vital Sign (NVS), which is a validated health literacy screener offered in English and Spanish. We additionally conducted focus groups of interdisciplinary PICU providers to identify factors which may influence implementation of health literacy screening using the Consolidated Framework for Implementation Research (CFIR) framework.

**Results:**

Among 48 surveyed caregivers, 79% demonstrated adequate health literacy using the Newest Vital Sign screener. The majority of caregivers spoke English (96%), were mothers (85%), and identified as White (75%). 83% of caregivers were able to attend rounds at least once and 98% believed attending rounds was helpful. Within the PICU provider focus groups, there were 11 participants (3 attendings, 3 fellows, 2 nurse practitioners, 1 hospitalist, 2 research assistants). Focus group participants described facilitators and barriers to implementation, which were mapped to CFIR domains. Timing of screening and person administering screening were identified as modifiable factors to improve future implementation.

**Conclusion:**

We found the health literacy levels of PICU caregivers in our setting is similar to prior assessments of parental health literacy. Participation in morning rounds was helpful for developing understanding of their child's illness, regardless of health literacy status. Qualitative feedback from providers identified barriers across all CFIR domains, with timing of screening and person administering screening as modifiable factors to improve future implementation.

## Introduction

When considering patient health and wellbeing, health literacy is a major driver of outcomes. Health literacy is defined as “the degree to which individuals have the capacity to obtain, process, and understand basic health information and services needed to make appropriate health decisions” (1). Unfortunately, it is estimated that over one-third of the adult population in the United States has limited health literacy skills to navigate the health care system (2). Limited health literacy is associated with increased hospitalizations, emergency visits, health care cost, and mortality (3). These effects are exacerbated in elderly, minority, poor, and limited English proficiency groups, making health literacy a critical target to address health disparities (4). Children are additionally affected, with limited parental health literacy negatively impacting pediatric health outcomes (5).

The health literacy of caregivers of critically ill children is unknown. Prior studies suggest that limited health literacy contributes to risk of hospitalization (3, 5, 6) and are correlated with discharge readiness (7). The pediatric intensive care unit (PICU) poses challenges to caregivers, heightening the importance of health literacy. Often, patients are admitted with multiple and/or complex diagnoses to an environment with high acuity and rapid change. Under these conditions caregivers may experience high levels of stress, which can decrease health literacy. One single center study found that half of PICU caregivers failed to comprehend the diagnosis, prognosis or treatment of the patient (8). There are often additional procedures or surgeries needed, which require adequate health literacy levels for informed consent (9, 10). Furthermore, many patients are discharged from the PICU with new morbidity (11) and require adequate health literacy to navigate new medications and treatments, specialty visits, and continued care at home. Understanding the health literacy of caregivers of critically ill children is foundational to addressing gaps in care, improving outcomes for this high-risk population, and decreasing healthcare costs.

The purpose of this mixed-methods study is to quantitatively assess the health literacy levels of caregivers of children admitted to the PICU and qualitatively describe facilitators and barriers to implementing health literacy screening in the PICU from the provider perspective. We theorize that the health literacy of PICU caregivers is less than the general population, which may contribute to the development of critical illness.

## Materials and methods

This is a mixed-methods single-center study. This study was deemed exempt by our local Institutional Review Board (IRB 21-019553).

### Quantitative methods: assessing health literacy of PICU caregivers

Caregivers were screened using the Newest Vital Sign (NVS) (12), which is offered in English and Spanish, and assesses both literacy and numeracy by asking the caregiver to interpret a nutrition facts label. To our knowledge, there is no specific screener for caregivers of hospitalized pediatric patients. We chose the NVS because it has been validated in outpatient pediatric caregiver populations and utilized in multiple prior studies (13) as well as piloted in the perioperative setting at our institution. Moreover, it was shown to be more predictive of emergency department use outcomes and more sensitive in identifying limited health literacy in younger caregivers of children when compared to the Short Test of Functional Health Literacy in Adults (S-TOFHLA) (14). Our PICU is a large, 75-bed quaternary referral unit located in an urban environment. Caregivers of patients admitted to the PICU (between August 12, 2022 and March 31, 2023) were approached by research study staff to complete a survey which included the NVS screener. Caregivers were approached during business hours, after checking in with bedside nurse to ensure it was an appropriate time to approach. Personnel or providers who accompanied patients from a long-term care facility were excluded. Caregivers for whom English or Spanish is not the first language were excluded, as the NVS is only validated in these two languages. We obtained self-reported demographics (primary language, age, sex, highest level of education completed, race, ethnicity) and patient demographics (age, presence of chronic illness, and prior hospitalizations). We also asked if the caregiver attended daily rounds and if it was helpful, since family-centered rounds are a primary mechanism by which PICU providers communicate with families. The primary outcome was health literacy level, which is determined by the NVS score. The scores range from 0 to 6, based on how many of the six questions are answered correctly. A score of 0–1 suggests high likelihood (50% or more) of limited health literacy; 2–3 is suggestive of possible limited literacy, and 4–6 almost always indicates adequate literacy (12). For the purposes of this study, we dichotomized the primary outcome into “Limited Health Literacy” (score of 0–3) and “Adequate Health Literacy” (score of 4–6). The association between demographic characteristics and health literacy level was assessed using Fisher's exact test. Data analysis was completed using Stata 17 (College Station, TX: StataCorp LLC).

### Qualitative methods: describing PICU provider perspectives around health literacy and screening

To understand provider perspectives around facilitators and barriers to health literacy screening, we conducted three focus groups of PICU providers. We included clinicians (physicians, advanced practice providers and hospitalists) for whom the PICU is their primary setting (hence rotating resident physicians were excluded). We opted for focus group structure to efficiently gather diverse opinions and provide a space for participants to build upon each other's input. Participants were recruited via email and received $20 gift card as compensation. The focus group script was based on the Consolidated Framework for Implementation Research (CFIR) to identify factors influencing implementation of screening including (1) intervention characteristics (2) outer setting (3) inner setting (4) individual characteristics and (5) implementation process (15). CFIR is a widely used framework (16) to systematically evaluate factors within a given setting which may influence intervention effectiveness (17). We additionally asked providers how they informally assess the health literacy of caregivers, and any approaches they use if they suspect limited health literacy. Each focus group was conducted virtually and recorded with the informed consent of the participants; the sessions were 45–60 min in duration. The sessions were later transcribed and de-identified prior to analysis. Thematic analysis of the three transcripts was completed by the PI in order to develop the initial codebook. Using this codebook, a second reviewer independently coded the transcripts. Inter-rater reliability was reported using a kappa statistic. Any discrepancies in coding were later discussed by both reviewers and resolved. Final themes were discussed among reviewers and organized by CFIR domains. Thematic analysis was completed using NVivo 12 (QSR, Melbourne, Australia).

## Results

Of 329 total caregivers who were approached, there were 48 total survey respondents (13%) of which 79% had adequate health literacy while 21% had limited health literacy (Table 1). The majority of caregivers spoke English (96% English; 4% Spanish). Most of the caregivers who completed the survey were mothers (85%) with 13% being fathers and 2% Other. The breakdown of race/ethnicity was predominately Non-Hispanic White (75%), with 10% Black/African-American, 4% Asian, 2% Multi-Racial, and 8% Prefer to Self-Describe or Prefer Not to Answer. The highest level of education completed by caregivers ranged from elementary school (6%), high school (19%), college (38%) to graduate school (31%), with 6% preferring not to answer. Demographic characteristics associated with health literacy level (*p* < 0.05) were primary language, age, race, and education level. 58% of parents reported that their child suffered from chronic illness. 19% of children had never been admitted, 31% were admitted once before, 10% had been hospitalized 2–3 times before, and 40% had been hospitalized more than three times. 83% of caregivers were able to attend rounds at least once and 98% believed attending rounds was helpful. Participation in morning rounds was associated health literacy level (*p* = 0.012).

**Table 1:** Characteristics of PICU caregiver survey respondents by health literacy level^a^.

	All	Limited literacy	Adequate literacy	*p*-value
	*N* = 48	*N* = 10	*N* = 38	
Primary Language				**0** **.** **040**
English	46 (96%)	8 (80%)	38 (100%)	
Spanish	2 (4%)	2 (20%)	0 (0%)	
Gender				1.00
Female	42 (88%)	9 (90%)	33 (87%)	
Male	6 (13%)	1 (10%)	5 (13%)	
Age				**0**.**010**
18–29 year-old	10 (21%)	5 (50%)	5 (13%)	
30–39 year-old	19 (40%)	2 (20%)	17 (45%)	
40–49 year-old	11 (23%)	0 (0%)	11 (29%)	
>50 year-old	8 (17%)	3 (30%)	5 (13%)	
Race (self-identified)				**0**.**006**
White	36 (75%)	4 (40%)	32 (84%)	
Black/African American	5 (10%)	3 (30%)	2 (5%)	
Asian	2 (4%)	1 (10%)	1 (3%)	
Multi-racial	1 (2%)	0 (0%)	1 (3%)	
Prefer not to answer	2 (4%)	0 (0%)	2 (5%)	
Prefer to self-describe	2 (4%)	2 (20%)	0 (0%)	
Relationship to the child				0.29
Mother	41 (85%)	8 (80%)	33 (87%)	
Father	6 (13%)	1 (10%)	5 (13%)	
Other	1 (2%)	1 (10%)	0 (0%)	
What is the highest level of education you completed?				**0**.**044**
Graduate School	15 (31%)	1 (10%)	14 (37%)	
College	18 (38%)	3 (30%)	15 (39%)	
High school	9 (19%)	2 (20%)	7 (18%)	
Elementary school	3 (6%)	2 (20%)	1 (3%)	
Prefer not to answer	3 (6%)	2 (20%)	1 (3%)	
Presence of chronic illness				0.72
No	20 (42%)	5 (50%)	15 (39%)	
Yes	28 (58%)	5 (50%)	23 (61%)	
Has your child ever been hospitalized before?				0.23
Never	9 (19%)	3 (30%)	6 (16%)	
Once	15 (31%)	1 (10%)	14 (37%)	
2–3 times	5 (10%)	2 (20%)	3 (8%)	
More than 3 times	19 (40%)	4 (40%)	15 (39%)	
Have you participated in morning rounds?				**0**.**012**
Never	8 (17%)	5 (50%)	3 (8%)	
Once	6 (13%)	0 (0%)	6 (16%)	
2–3 times	13 (27%)	3 (30%)	10 (26%)	
More than 3 times	21 (44%)	2 (20%)	19 (50%)	
Does participating in morning rounds help you understand what is happening with your child?				1.00
Yes	39 (98%)	5 (100%)	34 (97%)	
Not sure	1 (3%)	0 (0%)	1 (3%)	

Bolded values indicate statistical significance at a *p*-value < 0.05.

^a^ Data presented *n* (%) for categorical measures. Comparisons completed using Fisher's exact test.

For the PICU provider focus groups, there were 11 participants (3 attendings, 3 fellows, 2 nurse practitioners, 1 hospitalist, 2 research assistants). Years of experience in the PICU ranged from less than one year to 30 years. The inter-rater reliability between the two coders was a mean kappa of 0.53 (18).

The thematic analysis was divided to (1) describe current informal assessment of health literacy by PICU providers (2) approaches to families with perceived low health literacy and (3) facilitators and barriers to implementation by CFIR domains (Table 2).

**Table 2:** Facilitators and barriers to health literacy screening categorized by Consolidated Framework for Implementation Research (CFIR) domain.

CFIR domain	Key elements	Facilitators	Barriers
Outer Setting	External Pressure	–	**Socioeconomic factors preventing parents from being at the bedside** *…it's socioeconomic factors that are playing into it…so if they [caregivers] are working multiple jobs and they can't be there all the time… it's a limitation factor that our schedule doesn't match up with the parents in the PICU.*
Inner Setting	Relative Priority	**Survey is most successful when respondents have time and no competing priorities** *I think any setting where the parent maybe has to wait … I think is a good opportunity to kind of give this paper and be like, hey, you have time like here you go.*	**The ICU is stressful and caregivers place higher priority on child's status relative to survey completion** *When we're approaching PICU… they're busy focusing on the child, so when we approach them with the survey, it's at the back of their mind*.
Individuals	Motivation of team members	–	**Desire not to stigmatize families** *I’m worried about a screening tool like the one you showed, simply labeling them as low literacy, medium literacy, high literacy, and that's sticking with them and sort of being an impediment to communication rather than a facilitator.*
	Motivation of families	**Motivation is high if caregivers have an interest in research** *They might have maybe more knowledge on research and how it is beneficial for us to perform research and to be able to like kind of improve our healthcare in the long run. So maybe they're more keen on doing it.*	**Motivation is low if low interest or distrust in research**
Intervention	Design	**Including additional context to why health literacy screening is important** *I think giving them more context about how healthy literacy can improve communication with physicians and caregivers…* **Inquiring about communication or learning preferences** *Something that the survey does not get at, but maybe should … is assessing learning styles of caregivers and how they would want their information presented…*	**Screener focuses on nutrition label, which has little relevance to ICU medicine** *When we hand them the paper, they look at it and they're like, “What does health literacy have to do with reading an ice cream label?* **Screener is visual whereas most communication in ICU is verbal** *Reading comprehension and math comprehension test may be generally true in certain settings. But most information that we are conveying in the PICU is verbal*
Process	Adapting	**Adapting time of approach to extend to nights/weekends to reach more families** *I feel like one time that would be optimal is during rounds …But also obviously weekends or maybe even just after hours when the work, the nine to five work shift, is over*. **Changing who administers survey so that it is an existing team member** *I definitely think like a familiar face would be probably the like the better way to get a higher response rate*	**Researcher is not a familiar part of the team** *I feel like sometimes the research assistant goes in and they don't know us, they don't really– they hear research and they're kind of like, oh, OK, we'll maybe do it or they'll just say no automatically or just say yes to like get rid of us and they don't do it.*
	Tailoring strategies	**Using QR code/electronic interface is easier for data collection** *Logistically for us as researchers, QR code is definitely more convenient*	**QR code is difficult if no phone or less technologically savvy** *For the parents I might think that paper might be like more accessible and easier because they it's right in front of them, they don't have to scan anything to get into the survey*

Italicized font indicates quotes from focus group participants.

### Informal assessment of health literacy

Overwhelmingly, providers described using verbal cues to informally assess the health literacy of caregivers. The complexity of language and/or the use of medical jargon by caregivers led to a perception of higher health literacy and often prompted providers to ask if the caregiver had a background in healthcare. Most providers first asked parents to describe their understanding of the child's illness: “Asking them what they understand not just as a way of seeing what their health literacy is…but actually reflecting on the words that they're using to describe [the illness].” Providers also indicated that repeated encounters in healthcare, or even a few days of PICU admission, seemed to correspond with higher health literacy: “If someone is admitted right off the streets to the PICU for the first time ever, [I] will score them low on health literacy, but once they’ve been there five days, that grows.”

### Approaches to caregivers with perceived low health literacy

When asked how they respond when they suspect a caregiver may have limited health literacy, providers responded with (1) simplifying language (2) using visual aids or analogies and (3) increasing time spent with families. Not surprisingly, providers reported using less jargon, even if the caregiver has a medical background: “I still try to stick with simple language. Even if someone is in medicine, they’re not necessarily in critical care.” Providers reported using analogies or visual aids (i.e., showing imaging or laboratory trends) to help facilitate understanding. Providers also reported spending more time with families: “I think some families, more than others, will need someone to go back afterwards to make sure that everything has been talked through whether that is because of the clinical situation or because it's all new to them, or because we are worried that there are challenges in understanding.”

### Facilitator and barriers to health literacy screening

Facilitators and barriers were organized by CFIR domains of (1) outer setting (2) inner setting (3) individual characteristics, (4) intervention characteristics and (5) implementation process (Table 2).

### Outer setting

The outer setting was defined as the institutional systems and forces within the larger community that may influence health literacy screening. There were no facilitators identified, however respondents commented on external socioeconomic factors that may prevent caregivers from being at the bedside, thus decreasing opportunities for screening and education. There was additional commentary about how mothers may be more likely to be at the bedside if the familial structure was such that the father was working during the day and mother was primarily responsible for childcare. Similarly, the times that parents are at the bedside may not align with when is best for research or clinical teams to approach families.

### Inner setting

The overarching theme of the inner setting (the PICU) was the relative priority of participating in research being much less than a caregiver's worry for their critically ill child. The stressful environment of the ICU can be a barrier for all research, but in particular for this study which did not directly affect the patient's clinical status (as compared to enrolling in a clinical trial). The theme of relative priority was especially highlighted by the research team members who experienced screening patient in both PICU and perioperative settings; they cited differences in the parental attitudes because there was less stress and fewer competing priorities when caregivers were in the perioperative waiting room.

### Individual characteristics

When evaluating individual characteristics and attitudes, the greatest barrier to screening from the PICU provider perspective was a desire to not unfairly label caregivers based on their scores, or to “test” them during a time of stress. Providers were worried that the caregivers may be labeled as “health illiterate” and that it would impede, rather than promote, good communication. Providers also did not want to add an additional task to parents when they were stressed and overwhelmed. Lastly, as cited earlier, many providers assumed low health literacy in all caregivers and presumptively tailored their communication into smaller and simpler terms.

Regarding attitudes of caregivers, the success of screening seemed to be related to caregivers' perception of research. Some parents expressed an interest in research and knowledge of how participating in research can be helpful; other caregivers seemed to immediately dismiss researchers because they were not part of the care team or involved in direct patient care.

### Intervention characteristics

The most cited barrier of the screener itself was that the content of the screener (interpreting a nutrition label) seemed disconnected from PICU medicine and their child's illness. Parents might say it “[felt] like kindergarten math” or did not understand how a nutrition label could be related to health literacy. PICU providers felt that the interpretation of a nutrition label did not necessarily correspond with the skills needed to navigate critical illness. For example, the screener focuses on reading and math comprehension, whereas most of the communication in the PICU is verbal. Facilitators to improve screening included adding extra context and explanation as to why the screening was being done, and perhaps linking it to how the team could tailor their communication and education to the family.

### Implementation process

The implementation process facilitators and barriers centered on adapting (1) timing of when families were approached and (2) the person who administered the screener.

Barriers to approaching families tended to be that parents were not at the bedside or that it was a time of high acuity/stress. For this reason, facilitators to overcome these barriers highlighted approaching caregivers during different points of the admission or different times of day/night. Providers also commented that it would be helpful if the person administering the screener was a familiar part of the care team, as opposed to a researcher. Lastly, there was feedback regarding the best modality to administer the screener. Currently the screener requires scanning a QR code, which is helpful for the data collection process. However, for some caregivers who either do not have a smartphone or are less technologically savvy, the QR code was a barrier to completion.

## Discussion

The health literacy levels of caregivers of critically ill children have not been previously characterized. In this study, we present the health literacy levels of PICU caregivers in our single center using a previously validated screening tool, while simultaneously seeking qualitative input from providers about their perceptions of caregiver health literacy and factors which may influence implementation of health literacy screening. While preliminary in nature, this study is novel because it incorporates both quantitative and qualitative data, in addition to describing the health literacy levels of caregivers of critically ill children.

Overall, 79% of PICU caregivers demonstrated adequate health literacy which is comparable (if not higher) to the 72% that is reported for parents in the literature (19). Within our limited sample, demographic characteristics associated with health literacy level were primary language, age, race, and education level. These findings are consistent with prior literature showing that younger age, non-White race and lower education level are associated with limited health literacy (20). The demographics of the respondents were predominately English speaking mothers, potentially reflecting the characteristics of who is able to be present at the bedside or who is willing to complete the survey. One previous study showed that mothers may be more able to be present at the bedside in the PICU compared to fathers (21); the reason for this is unclear, but may be influenced by familial structure, employment status, and socioeconomic position. The racial/ethnic breakdown of respondents was predominately White. While the community in the immediate vicinity of our hospital is predominately Black, as a quaternary center which receives referrals from a larger, predominately White catchment area, there is a larger proportion of PICU patients which identify as White. The racial composition of our sample may simply reflect this trend, or as above, may be reflective of other factors allowing the parent to be at the beside. It may also be indicative of increased availability, trust, and willingness to participate in research. Qualitatively it seemed that attitudes around research contributed to survey completion and certainly prior studies have shown disparate participation by race based on different rates of approach, consent and/or distrust in research or healthcare system (22).

Interestingly, many parents (58%) reported that their child had a chronic illness and half the patients had been admitted more than once. Qualitatively, this corresponded to provider comments that caregivers' ability to use medical jargon increased if the patient had been admitted before—this raised the question of whether prior encounters with the healthcare system increase health literacy. Thus far the literature shows the opposite—parents of children with chronic illness and low health literacy showed decreased disease-specific knowledge (6, 23), decreased medication adherence (24), increased medication errors (25), and increased hospitalization (6). As one focus group participant said, if encounters with the health care system increased health literacy, then perhaps “the patient wouldn’t be in the PICU in the first place.” It may be, in fact, that the repeated encounters with the health care system are a product of low health literacy. Regardless, we did find that daily rounds enhance caregivers' understanding of their child's illness. The majority (83%) of screened parents were able to attend daily rounds at least once, with 98% reporting that it was helpful in understanding their child's illness. This positive impact of attending daily rounds has been shown repeatedly in prior studies (26–28).

Barriers to health literacy screening in the PICU spanned all CFIR domains, with emphasis on inner setting and individual characteristics. The inner setting of the PICU is fraught with stress and competing priorities which prevented some caregivers from completing the screener. Additionally, the attitudes of caregivers around research in general may impede successful screening. For some parents, participating in a research study that does not directly impact the trajectory of their child's illness is of low priority; others may not value or trust research *a priori*, which may contribute to decreased participation. Interestingly, providers' individual attitudes illustrated reluctance to “label” families as a barrier to screening. Instead, they seemed to favor either assuming low health literacy in all caregivers or asking about communication and learning preferences from caregivers.

Qualitative analysis of facilitators suggested that adapting the timing of screening as well as who performs the screening could improve implementation. Currently the screener is administered by research staff, however it may be more successful if administered by a care team member who is familiar to the caregivers. It may also decrease the perceived disconnect between screener and the child's PICU course by having an existing provider administer the screen. Regarding timing, it would be helpful to screen at night and during weekends in order to capture caregivers who cannot be at the bedside during business hours. There were also suggestions to approach at times when family is waiting (i.e., for transfer or discharge). We acknowledge that health literacy is dynamic and may change during the hospitalization; for example, it may decrease during times of high stress or increase with duration of hospitalization. While it may be less useful to screen at time of transfer/discharge because the bulk of the care has occurred, it could be argued as optimal to assess health literacy prior to transfer or discharge since it would alert the care team of a family who may require additional education or support prior to leaving the hospital.

There are many limitations to this exploratory study including screener characteristics, low response rate and subsequent small sample size, limited diversity in respondents, single center nature, and modest interrater reliability. The NVS has many advantages, including brevity, availability in Spanish, and validation in outpatient pediatric caregivers. However, as described by our focus group participants, the use of a nutrition label seemed less relevant in the PICU setting and seemed to confuse caregivers. Despite this, in the absence of a validated PICU-specific screener, we felt the NVS was the most appropriate choice. This study is also limited by small sample size of those who completed the survey, despite a much larger group being approached. As described in the qualitative feedback, there were many barriers to screening including external caregiver obligations, competing priorities, timing of screening, and preference for care team member to administer screening. These contributed to a low response rate and small sample size. There was also limited racial diversity in among study participants, with most identifying as White in the study relative to racial composition of our PICU population. Since all PICU patients were approached, we did not attribute this to differential approach for research, but potentially due to (1) differences in who is able to be present at the bedside during business hours and (2) differences in willingness to participate in research. To obtain a more diverse sample in the future, screening at night as well as utilizing a stratified sampling approach would strengthen screening efforts. Along these lines, we identified barriers and facilitators to researchers administering the NVS, which may be different if clinicians administered the screener. While ideally we would identify barriers/facilitators to clinician screening, we hoped this initial contextual inquiry of our unique PICU setting would help inform future efforts. The single center nature of the study is an important limitation; the health literacy of caregivers at a quaternary referral center may be different than a community PICU, highlighting the importance of multi-center studies to better understand the health literacy levels of these caregivers. Our qualitative findings, while providing important PICU provider insight, is limited by modest interrater reliability. Lastly, this study focused on barriers and facilitators from the provider/researcher perspective, however an important next step is to obtain qualitative feedback from parents and families. Not only could this identify unforeseen barriers and facilitators, but could illuminate additional ways in which providers can help support caregivers' understanding. The perspective of both providers and families could then be used to map implementation strategies to identify and support caregivers with limited health literacy.

Identifying caregivers with limited health literacy has important implications for how clinicians deliver information, educate, and support families during their admission and beyond. Strategies such as simplifying language, limiting information, using written and/or pictographic aids, and teach-back methods can be increased in clinician practice to promote patient understanding (29). Additionally, as more PICUs are implementing follow-up clinics for high risk patients (30), health literacy screening is a potential mechanism to identify patients for whom post-PICU follow up would be beneficial. Certainly future research, with larger samples, more diverse participation, and caregiver input, is needed to understand the health literacy of PICU caregivers and how PICU clinicians can better support patients and families.

## Conclusion

We found the health literacy levels of PICU caregivers in our setting are similar to prior assessments of parental health literacy. Participation in morning rounds was helpful for promoting caregivers' understanding of their child's illness, regardless of health literacy level. Qualitative feedback from providers identified barriers across all CFIR domains, with timing of screening and person administering screening as modifiable factors to improve implementation. Future efforts with larger samples, increased diversity of both participants and PICU settings, and qualitative perspectives from caregivers are needed to further characterize the health literacy levels of caregivers of critically ill children.

## Data Availability

The original contributions presented in the study are included in the article/Supplementary Material, further inquiries can be directed to the corresponding author.
